# P63 – Some unusual asthma triggers in tropical climes: an evidence-based review

**DOI:** 10.1186/2045-7022-4-S1-P118

**Published:** 2014-02-28

**Authors:** Chandramani Thuraisingham, Davendralingam Sinniah

**Affiliations:** 1 International Medical University, Seremban, Malaysia

## Introduction

Malaysia, a multi-racial country embraces different religious practices and customs. In this bazaar, we identified some unique asthma triggers that need sharing with other doctors. Awareness of these triggers can lead to better prevention and management.

## Objectives

This study reviews the evidence incriminating chichak, frankincense and myrrh, durian, ascaris, and spices as asthma triggers.

## Methods

A search was carried on these agents’ relationship to asthma using Google Scholar.

## Results

Chichak (hemidactylus frenatus). In tropical countries, chichak droppings accumulate behind furniture, pictures, and along walls. The faeces may contain salmonella27 and crypto-sporidiosis. Over time fungi growing on it liberate spores into the air causing allergies and asthma exacerbation. Frankincense and myrrh. Arabian incense burning is a known common asthma trigger among asthmatic Omani children. A report in the Bethlehem Journal of Medicine reveals that can cause ailments including asthma. Durian. This fruit known to “taste like heaven but smells like hell” is revolting and may cause anaphylaxis, but has not been proven to cause asthma. Ascaris. A large study on the interrelationships between asthma, atopy and helminthic infection in children from asthmatic families in rural China has shown that Ascariasis is closely associated with increased risk of childhood asthma, increased airway responsiveness to methacholine and was independent of sensitization to common aeroallergens. Spices. With globalization, curry is popular internationally. Capsaicin causes bronchoconstriction. As authentic curries are not tolerated by all, restaurateurs add tatrazine, a well-known trigger for asthma.

## Conclusion

There is adequate objective evidence to label chichak, frankincense, ascaris and spices, but not durian as asthma triggers. Preventive measures can reduce acute asthmatic attacks in sensitive individuals.

